# Fever as a first manifestation of acute aortic dissection

**DOI:** 10.1007/s12471-017-1025-9

**Published:** 2017-07-26

**Authors:** E. E. Siniorakis, S. G. Arvanitakis, A. G. Balanis, S. J. Limberi

**Affiliations:** Department of Cardiology, Sotiria Chest Diseases Hospital, Athens, Greece

To the Editor,

We enjoyed the recently published article by Jansen Klomp and colleagues [1], concerning the pitfalls in the differential diagnosis of acute aortic dissection (AAD). In their cohort, the authors report a high percentage of initially missed diagnoses, increased to 31%. Atypical symptoms and signs disorientate physicians during diagnosis.

We would like to focus on the importance of fever, an often overlooked sign, which may accompany AAD. Although rarely described among the classic manifestations of AAD, pyrexia is observed in one-third of cases, frequently as a first sign, especially in cases of painless dissections. Fever development may start with rigor, either simultaneously with AAD or preceding it by several days. Patients frequently present with pyrexia of unknown origin. It is not uncommon for fever accompanying painless AAD to be erroneously attributed to respiratory infections, leading to empirical treatment with antibiotics. Body temperature is often above 39 °C and left pleural effusion on chest X‑ray is frequently seen. Left-sided effusions are due to irritation of the left pleura from its contact with the aorta, or even to leakage from the dissection per se [2]. Pericardial effusions are also frequent in this setting. Although the white cell count, erythrocyte sedimentation rate and C‑reactive protein values are raised in febrile AAD, procalcitonin values remain normal, excluding an infective aetiology.

Pyrexia in the course of AAD is an ominous sign, related to high morbidity and mortality. Multi-arterial dissection is the rule, while death is the result of aortic rupture or multi-organ ischaemia and failure. As far as the origin of AAD-related fever is concerned, it seems that the formation of the pseudo-lumen recalls macrophages in the adventitia, with release of metalloproteinases and interleukin-6, which trigger a febrile immune-inflammatory mechanism [3].

It would be interesting to have some information about pyrexia in the cohort described by Jansen Klomp et al. [1]. Monitoring body temperature in patients fulfilling the criteria of potential AAD could develop into a reliable procedure of considerable diagnostic and prognostic value.
